# Aducanumab for Alzheimer’s disease?

**DOI:** 10.1136/bmj.n1682

**Published:** 2021-07-05

**Authors:** Sebastian Walsh, Richard Merrick, Richard Milne, Carol Brayne

**Affiliations:** 1 Cambridge Public Health, University of Cambridge, Cambridge, UK; 2 Society and Ethics Research Group at Wellcome Connecting Science, Cambridge, UK

## Abstract

Patients and families need hope, not false hope

The US licensing of Biogen’s aducanumab as “the first ever disease modifying drug for Alzheimer’s disease” was hailed as a major advance by many. However, in response to the decision, three members of the Food and Drug Administration’s expert independent advisory committee, which voted almost unanimously against approval, resigned, with Harvard professor of medicine Aaron Kesselheim describing it as “probably the worst drug approval decision in recent US history.”^1^ Given that existing treatments for Alzheimer’s disease have only marginal benefit at best,^2^ what does aducanumab’s controversial approval in the US mean for patients, clinicians, and researchers?

Amyloid protein clumps in the brain (plaques) are a neuropathological feature of Alzheimer’s disease and widely assumed to trigger a cascade of changes that cause cognitive decline. Aducanumab is a monoclonal antibody that removes amyloid plaques.^3^ The central controversy is whether the amyloid clearance protects patients from cognitive and functional decline.

This should have been answered by two identically designed (pre-approval) phase III trials, but it wasn’t. Both were stopped after preplanned early analyses on data up to December 2018 determined that the trials were “futile” (<20% chance of overall trial returning a positive finding).^4^ However, Biogen, which funded the trial, continued collecting data until the announcement of termination in March 2019. Reanalysis of data up to March 2019 confirmed the drug’s ineffectiveness in one study, but the other suggested cognitive benefit.

Biogen submitted its reanalysis to the FDA, and together they ran several retrospective analyses to explore the discrepancy between the two trials.^4^ Despite extensive evidence of ineffectiveness of other anti-amyloid agents,^5^ ^6^ the new analyses focused on explaining why one of the trials had returned a negative result, rather than exploring why the other one had not. None of these analyses found anything more persuasive than a chance result that would have “regressed to the mean” (averaged out as ineffective) had the trials continued to completion.^4^ ^7^

The FDA concluded that there were “residual uncertainties regarding clinical benefit.”^8^ Instead of recommending a new phase III trial, it granted a licence under its “accelerated approval” pathway for drugs that “may provide meaningful therapeutic benefit” based on a surrogate endpoint “that is reasonably likely to predict a clinical benefit.”^8^ This decision was remarkable because the only evidence that amyloid removal (a surrogate) slows cognitive decline (clinical benefit) comes from their retrospective analysis of the single trial and ignores abundant evidence of no benefit,^5^ ^6^ including the negative, identically designed trial.

## Years of uncertainty

Attempting reassurance, the FDA committed Biogen to a nine year post-approval confirmatory study. So we may not know until at least 2030 whether aducanumab slows cognitive decline, during which time the drug will be sold for use at a cost of $56 000 (£41 000; €43 000) per person each year.^1^ Moreover, phase IV post-approval trials may not be able to establish efficacy or lack thereof since, unlike pre-approval trials, they are designed primarily to identify rare side effects and real world effectiveness. We have been here before: the dementia drugs donepezil, galantamine, rivastigmine, and memantine were defunded in France in 2018 after over a decade of use because there was no evidence of clinically meaningful benefit.^2^

A big challenge for US clinicians and patients is the FDA decision to approve aducanumab for any patient with Alzheimer’s disease,^9^ despite Biogen’s trials including only those with early disease.

What will happen outside the US? In 2018 the European Medicines Agency (including the UK) updated its guidelines on clinical trials for Alzheimer’s disease^10^ to emphasise the need for trials to show cognitive and functional benefits rather than focusing solely on surrogate endpoints such as amyloid plaques. Approval of aducanumab in Europe would be inconsistent with this guidance and is therefore unlikely.

Even if approved, bodies such as the UK National Institute for Health and Care Excellence would struggle to reconcile uncertain clinical efficacy with the cost of treatment: as well as monthly intravenous infusions for an indefinite period, patients require repeated magnetic resonance imaging to monitor for side effects; 35% of patients in the trials experienced brain oedema and 19% micro-haemorrhages at the recommended dose.^4^

US approval of aducanumab has consequences for trials of other potential Alzheimer’s treatments. Researchers will now have to decide whether to use aducanumab or placebo as a control intervention. Use of placebo controls will be particularly challenging in the US when an FDA approved drug is already available.

This evolving story may ultimately damage public trust in regulatory and licensing institutions. This is deeply undesirable at any time, but particularly in the middle of a pandemic when public trust in lifesaving vaccines is so imperative.

People with Alzheimer’s disease and their families need hope, not false hope. Aducanumab’s approval on a technicality could undermine regulatory standards designed to protect against false hope and “set a dangerous precedent.”^11^ ^12^

The debate about the role of amyloid in Alzheimer’s disease remains intensely controversial. Aducanumab’s approval does little to resolve this controversy, while creating unhelpful uncertainties for patients, clinicians, and researchers. Some see aducanumab as proof of concept for the amyloid cascade theory, justifying decades of unsuccessful research costing billions of pounds and exposing thousands of participants to the side effects of experimental treatments. Others fear it will simply encourage futile investment in anti-amyloid therapies, diverting funds away from effective prevention measures such as improving physical activity or reducing hypertension,^13^ and better support after diagnosis.
